# Clinicopathological significance of c-MET and HER2 altered expression in bladder cancer

**DOI:** 10.1186/s43046-024-00250-2

**Published:** 2024-12-26

**Authors:** Engy Mohammed Naguib, EF Ismail, DI Badran, MH Sherief, TB El-Abaseri

**Affiliations:** 1 Medical Biochemistry and Molecular Biology Department, Faculty of Medicine, Suez Canal University, Ismailia, Egypt; 2 Urology Department, Faculty of Medicine, Suez Canal University, Ismailia, Egypt

**Keywords:** Bladder cancer, RT-PCR, *c-MET*, *HER2*, Gene expression

## Abstract

**Background:**

Tumor recurrence or metastasis after surgery is a significant factor influencing bladder cancer (BC) prognosis. Novel molecular biomarkers are necessary to determine each patient’s specific outcome because current biomarkers have limited power for predicting prognosis. The proto-oncogene MET encodes c-MET, a tyrosine kinase receptor. When c-MET attaches to its ligand, it triggers several steps in the signal transduction cascade that control cell survival, proliferation, and invasion. *c-MET* is overexpressed in several carcinomas. The HER2 gene encodes another receptor tyrosine kinase (RTK). *HER2* overexpression is linked to altered proliferation and increased aggressiveness in several malignancies. Identifying crosstalk partners of RTKs implicated in bladder cancer development may have a unique role in predicting aggressiveness. This study explored the expression status of c-MET and HER2 in human BC and their clinical significance in disease outcomes.

**Methods:**

A quantitative real-time polymerase chain reaction was done on 40 BC patients who had undergone transurethral resection (TUR) or radical cystectomy and had a pathologically verified diagnosis of primary tumor without prior chemoradiotherapy as well as 20 patients with benign diseases who served as controls. The c-MET and HER2 expression levels were investigated, and their relationship with clinicopathological features was analyzed.

**Results:**

*c-MET* and *HER2* gene expression were significantly higher, 6.1- and 4.5-fold, in the study group compared to the controls. The frequency of c-MET and HER2 overexpression in the study group was 80% (32/40) and 90% (36/40), respectively. *c-MET* overexpression was associated with pathological stage(*P* = 0.002), tumor grade (*P* = 0.019), muscle invasion (*P* = 0.008), and node involvement (*P* = 0.017), while *HER2* overexpression was associated with pathological stage(*P* = 0.033), invasion to muscles (*P* = 0.003), and node involvement (*P* = 0.005). Based on the Log-rank test, patients expressing both *c-MET* and *HER2* had the poorest disease-free survival rates among all studied patients (median = 10 m, 3.0–16.9 95%CI).

**Conclusion:**

There is a possible correlation between c-MET and HER2 gene overexpression and poor clinical outcomes in patients with BC.

## Introduction

Bladder cancer (BC) ranks as the 6th most common cancer in men and the 17th most common in women globally. In Egypt, BC is the 3rd most frequent malignancy, following liver and breast cancer [1]. Urothelial carcinoma is the most common type of BC, comprising around 90% of cases in industrialized countries and 80% in other parts of the world. Among the many diverse BC subtypes, the common non-muscle invasive bladder cancer (NMIBC) is linked to a promising prognosis. In contrast, the less frequent muscle-invasive bladder cancer (MIBC) is linked to a bad prognosis [2]. About 75–85% of individuals have NMIBC; the usual treatment is transurethral resection (TUR). Approximately 60–70% of these tumors recur, with 25% progressing to a higher stage or grade. The significant recurrence rate following surgery suggests that this treatment may not be optimum for all patients [3].

Cellular mesenchymal-epithelial transition factor (c-MET) is included in the tyrosine kinases receptor superfamily and is coded by the proto-oncogene MET. c-MET interacts with its ligand, hepatocyte growth factor (HGF), activating several steps in the signal transduction pathway that control the survival of cells, their proliferation, and invasion. *c-MET* is upregulated in various cancers, including lung, breast, ovarian, gastric, and kidney. It is related to epithelial-to-mesenchymal transition, muscle invasion, and poor long-term survival [4].

The human epidermal growth factor receptor 2 (HER2) gene codes another RTK termed HER2. HER2 activity is vital for proper cellular growth, and its upregulation is associated with abnormal proliferation and enhanced aggressiveness in numerous cancers, including breast, bladder, colon, and lung [5].

Increasing data suggests that abnormally activated RTKs may not operate in isolation but rather interact with other pathways in healthy and altered cells, a phenomenon known as (RTK co-activation) [6]. The crosstalk between c-MET and HER2 was previously reported to drive aggressive lung carcinoma by maintaining signaling via the PI3K-AKT and MAPK pathways [7]. Gastric cancer patients expressing both RTKs tended to have lower overall survival rates. The interaction between c-MET and HER2 was linked to the therapeutic resistance of several cancers, including breast, lung, and esophagogastric cancer [8]. Although the possible interplay between c-MET and HER2 in BC was not fully elucidated, exploring the c-MET and HER2 possible interplay could offer valuable insights into identifying novel biomarkers for BC aggressiveness.

## Methods

### Ethical approval and informed consent

The study adhered to the Declaration of Helsinki and received approval from the Medical Research Ethics Committee of Suez Canal University’s Faculty of Medicine (Approval No. 3653). All participants gave written consent.

### Study groups and collection of specimens

The study comprised 60 patients with complete medical records from Suez Canal University Hospital enrolled in the Urology department between January 2020 and December 2020. Following informed consent, tissue samples from original tumors were taken from 40 patients who had transurethral resection (TUR) or radical cystectomy and were confirmed histopathologically as urothelial carcinoma. As a control, 20 patients with non-cancerous conditions, including stones, cystitis, or urinary incontinence, had their bladder mucosa extracted. All specimens were fixed in neutral buffered formalin and processed to embed paraffin. The formalin-fixed paraffin-embedded (FFPE) tissue blocks were cut into sections, 4-μm thick, and stained with hematoxylin and eosin (H&E) for histopathological re-evaluation by two independent pathologists. All cases were re-evaluated, graded, and staged using the WHO 2016 classification and the TNM staging approach [9].

### Deparaffinization of FFPE tissue and total RNA isolation

Total RNA was isolated from 5- to 20 μm-thick sections using the xylene and RNeasy® FFPE kit Qiagen (cat. no. 73504), according to the manufacturer’s instructions. The RNA’s quality and integrity were validated using a Nanodrop ND-1000 spectrophotometer (NanoDrop Tech., Inc., Wilmington, DE, USA).

### C-MET and HER2 gene expression analysis

Qiagen’s Quantitect Reverse Transcription Kit (Cat. no. 205311) generated complementary DNA (cDNA) from 1 μg of total RNA. PCR was carried out using an ABI StepOne device and SYBR Green hot-start master mix (QuantiNova SYBER Green PCR Kit, cat. no.208054, Qiagen) according to the manufacturer’s instructions. The relative quantification approach was used to compute specific mRNA levels and adjust them to GAPDH, a housekeeping gene [10].

Primers for gene expression analysis were created using the online Primer Quest tool (Integrated DNA Technologies), employing published sequences from the NCBI GenBank database. They were double-checked via in-silico PCR amplification with the NCBI primer blast tool. The primers are provided in Table 1.

**Table 1:** Primer’s sequence

Gene	Primer sequence
*c-MET*	Forward 5′-CATGCCGACAAGTGCAGTA-3′, reverse 5′-TCTTGCCATCATTGTCCAAC-3′,
*HER2*	Forward 5′- ACCAGTCCAAAGTCCCTCCTT3′, reverse 5′-GAAGGTGTCAGTTCCGGCTT-3′
*GAPDH*	Forward 5′-TCCAAAATCAAGTGGGGCGA-3′, reverse 5′-GCCTTCCTCACCTGATGATCT-3′

Data was analyzed using the Applied Biosystems software. Real-time PCR followed the Minimum Information for Publication of Quantitative Real-Time PCR Experiments guidelines [11].

### Statistical analysis

The data was processed with SPSS for Windows version 22 and R version 3.3.1 (corrplot, dplyr, and ggplot2 tools). Since the data was not normally distributed, we used the Mann–Whitney *U* and Kruskal–Wallis tests to compare it. A two-tailed significance level < 0.05 indicated statistical significance. The Livak technique was utilized to calculate the fold change of c-MET and HER2 in all cancer patients against control patients using the threshold cycle (CT) value. Kaplan–Meier curves were used to estimate patients’ disease-free survival rates (DFS).

## Results

### Sociodemographic data of the studied subjects

The mean age (± SEM) of subjects in the patients’ group (66.9 ± 1.6) was matched with the controls (63.7 ± 1.6). Seventy-three (92.5%) of the patients’ group were males and 3 (7.5%) were females, while in the controls, 16 (80%) were males and 4 (20%) were females. Table 2 illustrates the BC patients’ baseline features.

**Table 2:** Clinicopathological characteristics of the BC patients

Variables	Frequency (%)	
Age (year)	** < 70**	**22 (55%)**
	** ≥ 70**	**18 (45%)**
Tumor size (cm)	** < 3 cm**	**18 (45%)**
	** ≥ 3 cm**	**22 (55%)**
Grade	**Low**	**14 (35%)**
	**High**	**26 (65%)**
Pathological stage	**Tis**	**––**
	**Ta**	**12 (30%)**
	**T1**	**15 (37.5%)**
	**T2**	**6 (15%)**
	**T3**	**7 (17.5%)**
Muscle invasion	**-**	**31 (77.5%)**
	** + **	**9 (22.5%)**
LNS involvement	**-**	**34 (85%)**
	** + **	**6 (15%)**
Metastasis	**-**	**36 (90%)**
	** + **	**4 (10%)**
Multiplicity	**-**	**30 (75%)**
	** + **	**10 (25%)**

### The relative expression of c-MET and HER2 genes in studied groups

**Fig. 1:**
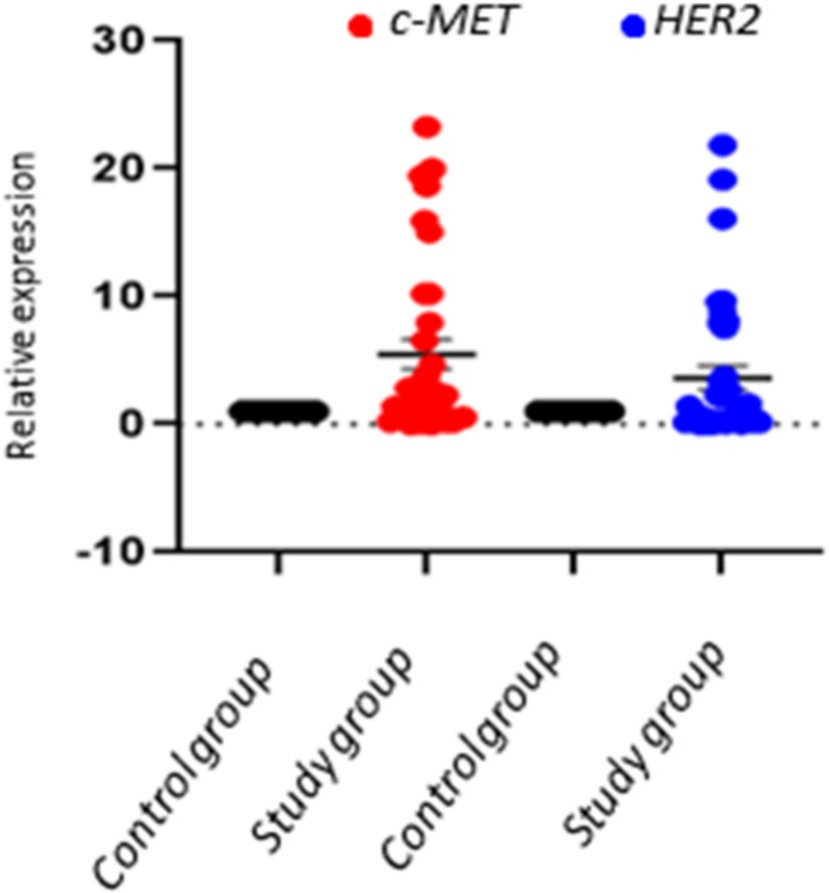
Relative expression of c-*MET* and *HER2* in BC. C-MET and *HER2* Expression were compared between the study group and controls by qRT-PCR. *GAPDH* was used as an internal control. Data is presented as a column scatter plot showing mean and SEM. Red dots represent each c-*MET* delta delta ct value, while blue dots represent *HER2* delta delta ct values

The mean expression of the c-MET gene was 6.1 times greater in malignant tissue than in noncancerous tissue. This distinction was statistically significant (*P* = 0.001), and the mean HER2 gene expression was 4.5-fold greater in malignant tissue than in noncancerous tissue (*P* = 0.004) (Fig. 1).

### Association between c-MET and HER2 genes expression and pathological features

Increased c*-MET* expression was linked to the pathological stage (*P* = 0.002), grade (*P* = 0.019), muscle invasion (*P* = 0.008), and node involvement (*P* = 0.017), while *HER2* expression was associated with the pathological stage (*P* = 0.033), muscle invasion (*P* = 0.003), and tumor grade (*P* = and node involvement (*P* = 0.005). We could not find significant differences between *c-MET* expression, gender, age, metastasis, tumor size, and multiplicity. Also, non-significant differences were observed between *HER2* expression, gender, age, tumor size, and metastasis (Table 3).

**Table 3:** Association between c-MET and HER2 genes expression and pathological features

Variables		Frequency (%)	c-MET gene expression Mean (SEM)	*P*-value	HER2 gene expression Mean (SEM)	*P*-value
Tumor size (cm)	< 1 cm	5 (12.5%)	33.5 (22.2)	0.584	20.1 (9.5)	0.450
	1–3 cm	13 (32.5%)	12.5 (7.6)		13.9 (7.3)	
	> 3 cm	22 (55%)	95.4 (18.9)		17.2 (5.7)	
Grade	Low	14 (35%)	5.1 (1.7)	0.019*	8.7 (3.9)	0.033*
	High	26 (65%)	52.9 (12.4)		21.2 (5.9)	
Pathological stage	Ta	12 (30%)	5.1 (1.9)	0.002*	6.4 (3.3)	0.014*
	T1	18 (45%)	34.2 (12.0)		9.9 (3.6)	
	T2	4 (10%)	80.1 (69.9)		36.9 (18.3)	
	T3	6 (15%)	106.6 (40.7)		42.8 (16.6)	
Muscle invasion	-	31 (77.5%)	21.8 (7.4)	0.008*	11.1 (3.5)	0.003*
	+	9 (22.5%)	106.4 (37.2)		35.4 (11.7)	
LNS involvement	-	34 (85%)	37.2 (13.1)	0.017*	11.9 (3.3)	0.005*
	+	6 (15%)	61.8 (14.6)		42.5 (16.7)	
Metastasis	-	36 (90%)	38.9 (12.4)	0.071	15.3 (4.3)	0.112
	+	4 (10%)	58.4 (18.2)		27.3 (11.8)	
Multiplicity	-	30 (75%)	52.6 (14.5)	0.158	21.2 (5.1)	0.012*
	+	10 (25%)	5.98 (2.2)		2.4 (1.1)	

^*^P values are based on Kruskal–Wallis or Mann–Whitney *U* tests as appropriate. *P* < 0.05

*SEM* standard error of the mean

### Correlation between the expression of c-MET and HER2 genes in the study group

**Fig. 2:**
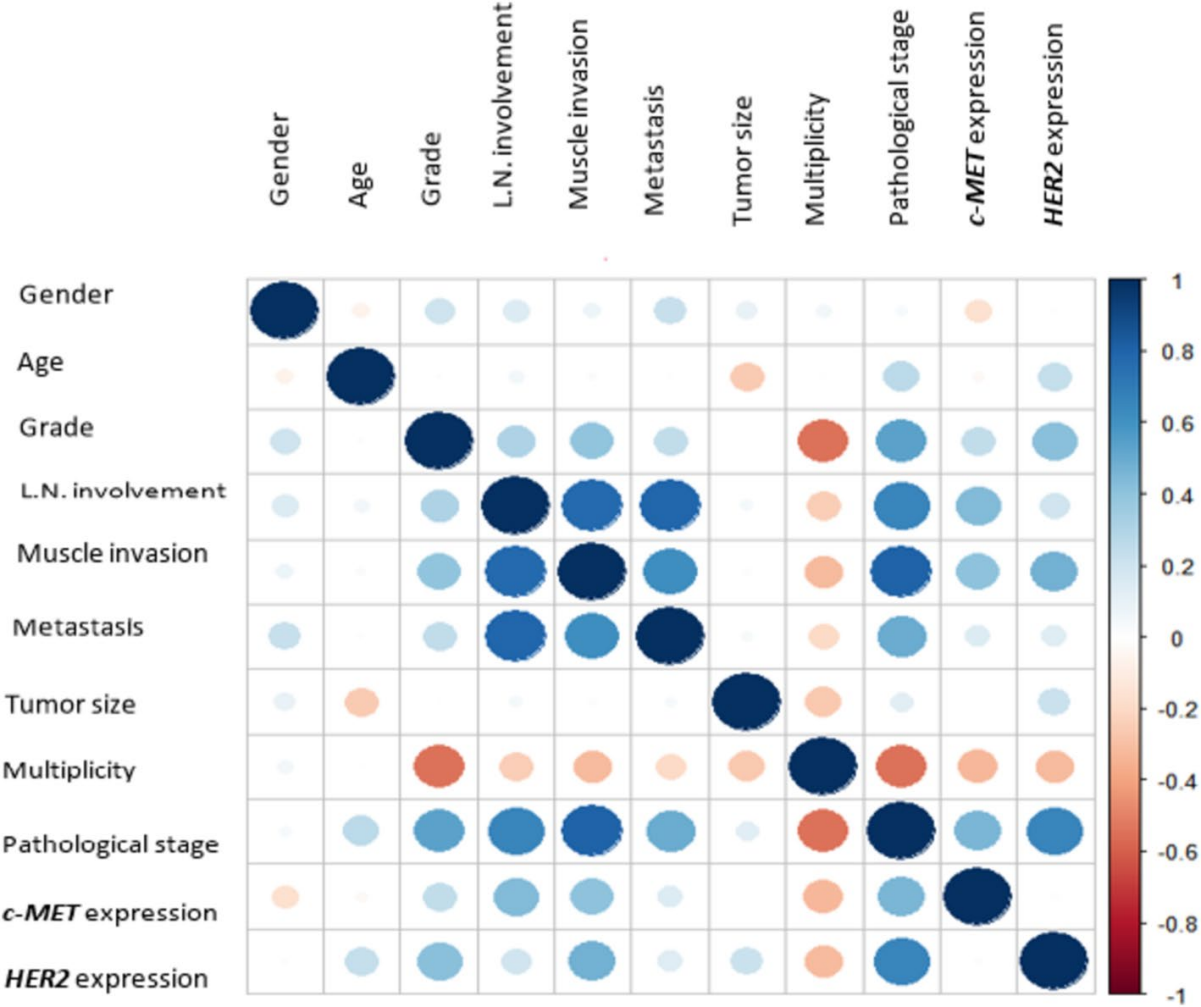
Correlation analyses between c-MET, HER2, and clinicopathological feature. Pearson’s correlation was computed with the corrplot R tool. The magnitude of the color on the right bar indicates the degree of correlation. The *r* value fluctuates between − 1 and 1. *r* = 0.6–0.8 indicates a substantial correlation. *r* = 0.4–0.6 suggests a strong connection; *r* = 0.2–0.4 shows a mild link, and *R* = 0 indicates no correlation. − values indicate inverse correlation, while + values represent positive correlation

Data analysis showed no correlation between *c-MET* and *HER2* expression: regarding *c-MET* expression, a moderate positive correlation with the pathological stage (*r* = 0.6) and a weak positive correlation (*r* < 0.5) with grade and muscle invasion, while *HER2* expression showed a weak positive correlation with muscle invasion, tumor stage, and lymph node involvement (*r* < 0.5) (Fig. 2).

### Expression of c-MET, HER2, and disease-free survival (DFS)

**Fig. 3:**
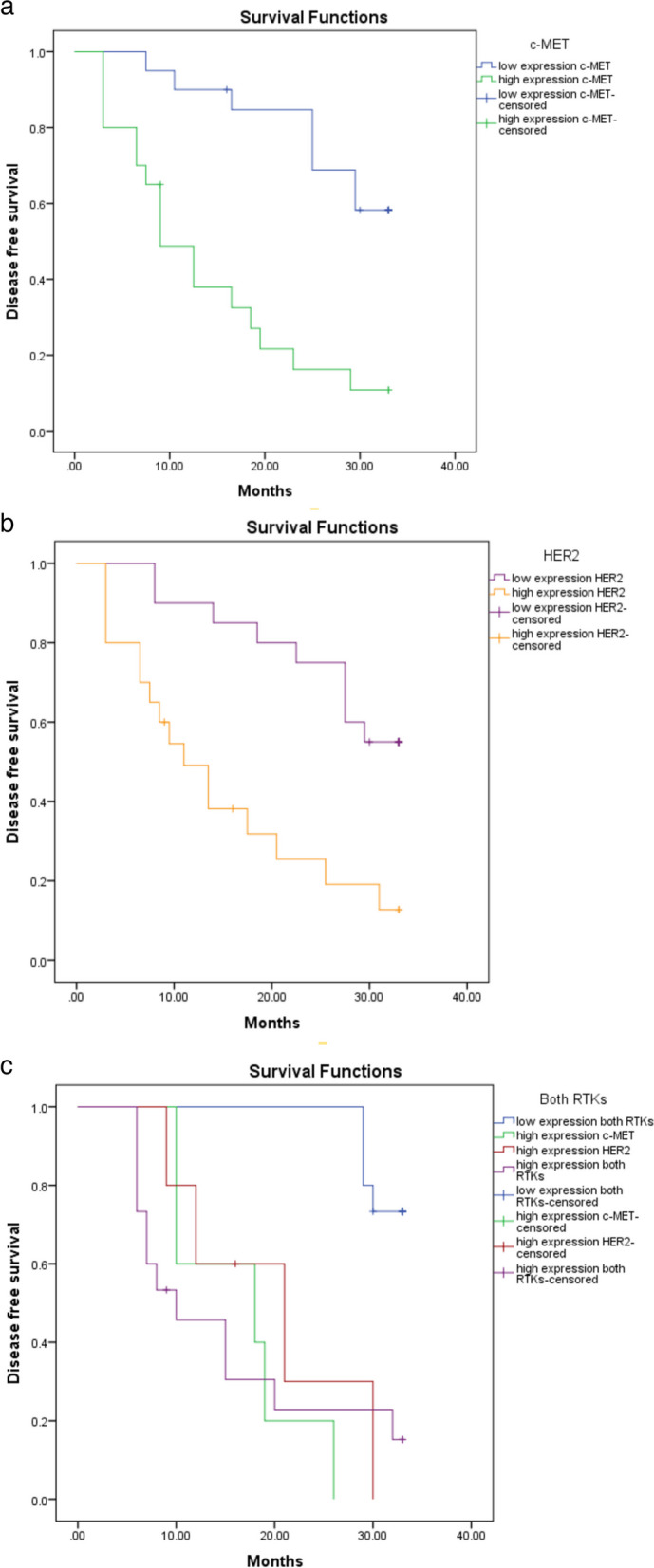
Disease-free survival curves grouped by RTK expression. Survival analysis according to* c-MET* (**A**), *HER2* (**B**), and both RTKs (**C**)

For BC patients (*n* = 40), after follow-up for 33 months, 25 patients (62.5%) experienced disease recurrence. Patients were categorized into high- and low-expression categories based on the median as cut points. Patients with high *c-MET* expression had poorer DFS compared to the low *c-MET* expression group (*P* = 0.0001) based on the log-rank test with a median survival of 10 months (4.2–15.7 95% CI); patients with low *HER2* expression seem to have more favorable survival than patients with high *HER2* expression group (median = 12 m, 5.8–18.1 95% CI) (*P* = 0.001). The log-rank test indicates a significant difference between the survival curve of patients highly expressing both RTKs (*c-MET* and *HER2*) and those with low expression for both RTKs (*P* = 0.001): patients with high expression of both RTKs had the poorest survival rate among all groups (median = 10 m, 3.0–16.9 95% CI) (Fig. 3).

## Discussion

Crosstalk between RTKs can promote tumor growth and metastasis, making them attractive therapeutic targets. Three basic mechanisms of RTK crosstalk have been identified: trans-phosphorylation and activation to produce heterodimeric receptor complexes that activate both tyrosine kinases, transcriptional activation of the ligand, and activation via metalloproteinases [12].

A recent study found that crosstalk between MET and HER2 maintains signaling resilience via cell survival effectors such as the PI3K-AKT and MAPK pathways, which drive aggressive squamous cell carcinoma [7]. Recent research has discovered interactions between the MET receptor and HER2 receptor families in the treatment resistance of numerous malignancies, including breast, lung, and esophagogastric cancer [13–15].

In the present study, we analyzed c-MET and HER2 genes to quantify the level of expression of each of the c-MET and HER2 genes in Egyptian BC patients and to determine whether simultaneous changes in the expression of both genes can be used to determine BC patients at a greater risk of developing aggressive disease, with the goal of better stratification and co-targeting therapy. Furthermore, the association between the expression of both genes and several disease phenotypes was investigated.

We found that the mean *c-MET* level was 6.1-fold higher in BC patients compared to controls, and such change was statistically significant. Similarly, Cheng et al. [16] investigated the MET receptor expression in bladder tissue using (IHC) in non-neoplastic urothelium with inflammatory illness and malignant patients. For BC, 4.9% of tumors showed high MET expression, 22.5% showed low expression, and 72.5% showed no expression. The basal cells of the mucosa showed weak and sporadic staining for nonneoplastic urothelium. Also, Kim et al. [17], a prior study, found that c-MET, AXL, and PDGFR overexpression enabled the differentiation of cancerous and non-cancerous tissues using q-PCR and predicted poorer prognosis in NMIBC patients.

In contrast to our finding, Kluth et al.’s [18] IHC and FISH analysis of tissue microarrays containing BC tissues revealed that membrane MET expression is frequent in both non-cancerous bladder and BC tissues. Kluth et al. discovered that lower levels of membranous MET were linked to a high-grade and more advanced tumor stage in BC. Unlike earlier investigations, the authors claimed to have evaluated a variety of commercially available antibodies [18].

Overexpression of *c-MET* could be due to transcriptional activation, hypoxia-induced overexpression, or amplification [4].

We found that the upregulation of *c-MET* was linked to muscle invasion (*P* = 0.008). The result presented herein agrees with previous research, which found that c-MET overexpression is associated with the invasion of muscles (*P* < 0.001) [4]. Also, Mukae et al. [19] studied c-MET in FFPE BC tissue by IHC and reported that HO-1 and PD-L1 possibly regulate the invasion of muscle.

C-MET overexpression activates several intracellular signaling pathways, promoting motility, invasiveness, and epithelial-mesenchymal transition (EMT), enhancing carcinoma cells’ invasive capacity [4].

Regarding *c-MET* and tumor grade, we found that *c-MET* expression was linked to tumor grade (*P* = 0.019). Cheng et al. [16] agreed with our result; MET was positively associated with tumor grade (*P* < 0.05). In contrast to our findings, Xu et al. [20], in their meta-analysis, which included 1336 cases, reported that c-MET overexpression did not correlate with BC grade. Kluth et al. [18] showed an inverse connection between MET expression and BC characteristics, including tumor grade. The discrepancy in results may be due to various techniques utilized for expression assay.

Our findings disclosed that higher *c-MET* levels are related to both pathological stage (*P* = 0.002) and lymph node (L.N.) involvement (*P* = 0.017). Miyata et al. [21] observed that phosphorylated MET (pY1234) was the most significant predictor for the high pathological stage, while c-MET was substantially related to the pathological stage. In contrast, Iyer et al. [22] discovered no significant correlation between c-MET immunoreactivity and pathogenic stage. Cheng et al. [16] observed that pY1234/1235 c-MET and pY1349 c-MET levels were linked to L.N. involvement. However, no significant relationships were detected for non-phosphorylated c-MET. In parallel, Yeh et al. [23] observed that c-MET expression did not correlate with L.N. involvement.

We could not find a significant association between *c-MET* expression, gender, age, metastasis, tumor size, and multiplicity. Previous research had similar findings [13–15].

In our investigation, HER2 expression was 4.5 times greater in patients with BC than in controls. Parallel to our findings, Gan et al. [24] did a meta-analysis of 1398 patients and used the TCGA database for research. At transcription and translation levels, HER2 expression was considerably greater in cancerous than non-cancerous tissue. In agreement with our data, Sanguedolce et al. [25] claimed that HER2 expression was considerably enhanced in BC, and this feature related to tumor growth.

HER2 upregulation in BC is primarily due to gene amplification, which activates pathways which enhance cell growth, migration, and aggressiveness. In a sample of BC patients, there was a very high incidence of chromosome 17 polysomy (97%) and a higher HER2 copy number (92%) [24].

We found that *HER2* level was connected to the grade of tumors and pathological stage of BC (*P* = 0.033, 0.014, respectively). Previous research has shown similar outcomes. In agreement with our study, Ahmed et al. [26], who assessed HER2 protein by IHC in BC tissues, reported a statistically significant variation in HER2 expression between high-grade and low-grade BC. Ahmed found that HER2 expression was directly related to the grade and stage of the tumor and may be a trigger for tumor progression in BC pathogenesis. Also, Kiss et al. [27] reported higher HER2 protein levels in 11.7% of patients, which were correlated with tumor grade (*P* = 0.003) and pathological stage (*P* = 0.015). Contradicting our findings, de Peneaux et al. [28], who analyzed archival biopsy specimens from transitional BC immunohistochemically for the expression of the HER2, found no correlation between HER2 protein overexpression and the grade and stage of BC.

Higher HER2 levels were associated with L.N. involvement in BC (*P* = 0.005). The previous study also mentioned that the upregulation of HER2 expression is related to BC lymph node invasion [27]. Consistent with us, Fleischmann et al. [29] demonstrated that the prevalence of HER2 protein reactivity in lymph node metastases was much greater than in the primary site of BC.

In our study, *HER2* expression was significantly associated with multifocal tumors (*P* = 0.012). A prior meta-analysis demonstrated that the HER2 protein tends to be significantly expressed in multifocal cancers [27].

Moreover, our data showed that HER*2* expression significantly correlates with muscle invasion (*P* = 0.003). In agreement with our findings, Esmail et al. [30] retrospectively studied paraffin blocks by immunostaining. HER2 expression was detected in 25% of NMIBC cases and 90% of MIBC cases, with *P* < 0.001.

We found no link between HER2 expression, gender, or age in BC patients, and multiple studies, either by immunohistochemistry or PCR, reported the same findings [27, 30].

Identification of crosstalk partners of RTKs involved in tumorigenesis is crucial as essential biomarkers for aggressiveness and co-targeting therapy. MET and HER2 have previously been shown to compensate for each other after targeted knockdown via upregulation, activation of the other RTK network, or compensatory ligand stimulation [12].

Our study’s correlation analysis between c-MET and HER2 gene expression showed no correlation (*r* = 0.019). This could be due to the small sample size; the interaction mechanism between c-MET and HER2 could be through activation of the other RTK or ligand stimulation, not only upregulation. Different detection techniques like IHC may help study the co-expression status of MET and HER2 and test the concordance of RT-qPCR results.

In contrast, Sanchez et al. [15] investigated c-MET, FGFR2, and HER2 expression levels in human gastric cancer (GC) and their therapeutic implications in clinical treatment. In the RTKs co-expression study, 3.5% of the patients tested triple positive. Patients with triple-positive GC had the shortest overall survival rates. In contrast to our findings, breast cancer has shown co-expression of MET and HER2, which has led to RTK inhibitor resistance via MET amplification or autocrine production of MET ligands [14].

Survival study indicated that patients highly expressing *c-MET* had worse (DFS) relative to the low c-MET expression group (*P* = 0.0001), and patients with high HER2 levels appear to have a worse DFS than patients with low HER2 expression (*P* = 0.001). We discovered that patients who expressed both RTKs (c-MET and HER2) had the lowest DFS rate of all patients. (*P* = 0.001). Similarly, Xu et al. discovered an elevated risk of poor overall survival (OS) related to elevated c-MET expression in BC patients. C-MET overexpression was an independent predictor for lower long-term survival [21]. Gan et al. [24] discovered that individuals with high HER2 protein expression had a higher risk of cancer recurrence (HR = 0.76; 95% CI: 0.63–0.92; *P* < 0.01) and progression compared to those with low HER2 levels. HER2 upregulation was linked to a lower 2-year recurrence-free survival (RFS) rate. In another study, amplification and overexpression of *HER2* were correlated with poor disease-specific survival (*P* = 0.02) [25].

The poor survival observed in patients with high expression of c-MET and HER2 could be explained by the possible crosstalk between both receptors. This could occur through several mechanisms, including forming heterodimer complexes, transactivation, or downstream signaling convergence. Both receptors trigger similar downstream signaling pathways, including PI3K/AKT and MAPK, leading to enhanced signaling and tumor progression [6].

## Conclusion

The study highlighted the association of *c-MET* and *HER2* overexpression with standard pathological markers of lousy prognosis in BC (pathological stage, muscle invasion, node involvement, and tumor grade). These findings suggest a possible correlation between *c-MET* and *HER2* overexpression and poor clinical outcomes in patients with BC.

## Data Availability

All the data are reported in the manuscript.

## Abbreviations

AKT: Protein kinase B
AXL: Receptor tyrosine kinase, derives its name from the Greek word Anexelekto
BC: Bladder cancer
c-MET: Mesenchymal-epithelial transition factor
COX-2: Cyclooxygenase-2
EGFR: Epidermal growth factor receptor
EMT: Epithelial-mesenchymal transition
ERK: Extracellular signal-related kinase
FAK: Focal adhesion kinase
FAS: Fas cell surface death receptor
GAB1: Growth factor receptor bound protein 2-associated protein 1
GRB2: Growth factor receptor-bound protein 2
HER2: Human epidermal growth factor receptor 2
HGF: Hepatocyte growth factor
HO-1: Heme oxygenase-1
JAK2: Janus tyrosine kinase 2
LOH: Loss of heterozygosity
MAPK: Mitogen-activated protein kinase
MIBC: Muscle-invasive bladder cancer
PD-L1: Programmed cell death ligand 1
PD: Programmed cell death
PI3K: Phosphatidylinositol 3 kinase
PIK3CA: Phosphatidylinositol-4,5-bisphosphate 3-kinase catalytic subunit alpha
RTKs: Receptor tyrosine kinases
SRC: Sarcoma
STAT: Signal transducer and activator of transcription
UC: Urothelial carcinoma
